# NGF and Its Receptors in the Regulation of Inflammatory Response

**DOI:** 10.3390/ijms18051028

**Published:** 2017-05-11

**Authors:** Gaetana Minnone, Fabrizio De Benedetti, Luisa Bracci-Laudiero

**Affiliations:** 1 Division of Rheumatology and Immuno-Rheumatology Research Laboratories, Bambino Gesù Children’s Hospital, 00146 Rome, Italy; gaetana.minnone@yahoo.it (G.M.); fabrizio.debenedetti@opbg.net (F.D.B.); 2 Institute of Translational Pharmacology, Consiglio Nazionale delle Ricerche (CNR), 00133 Rome, Italy

**Keywords:** sensory sympathetic and parasympathetic neurons, neuropeptides, neurotransmitters, innate immunity, inflammatory diseases

## Abstract

There is growing interest in the complex relationship between the nervous and immune systems and how its alteration can affect homeostasis and result in the development of inflammatory diseases. A key mediator in cross-talk between the two systems is nerve growth factor (NGF), which can influence both neuronal cell function and immune cell activity. The up-regulation of NGF described in inflamed tissues of many diseases can regulate innervation and neuronal activity of peripheral neurons, inducing the release of immune-active neuropeptides and neurotransmitters, but can also directly influence innate and adaptive immune responses. Expression of the NGF receptors tropomyosin receptor kinase A (TrkA) and p75 neurotrophin receptor (p75NTR) is dynamically regulated in immune cells, suggesting a varying requirement for NGF depending on their state of differentiation and functional activity. NGF has a variety of effects that can be either pro-inflammatory or anti-inflammatory. This apparent contradiction can be explained by considering NGF as part of an endogenous mechanism that, while activating immune responses, also activates pathways necessary to dampen the inflammatory response and limit tissue damage. Decreases in TrkA expression, such as that recently demonstrated in immune cells of arthritis patients, might prevent the activation by NGF of regulatory feed-back mechanisms, thus contributing to the development and maintenance of chronic inflammation.

## 1. Introduction

The discovery of nerve growth factor (NGF) more than 60 years ago is associated with embryonal development and the differentiation of peripheral neuronal cells. Studies by Levi-Montalcini in chick embryos clearly demonstrated that the production of this diffusible factor in the target organ was responsible for the survival of peripheral neurons during development. The purification of NGF, its biochemical characterization and the production of neutralizing antibodies made it possible to demonstrate in a number of animal models, both in vitro and in vivo, that NGF is essential for the survival of sympathetic and sensory neurons during nervous system differentiation [1]. The release of NGF in the innervation field not only guarantees the survival of neurons [2] but also regulates axon growth and synapse formation [3], in addition to influencing neurotransmitter and neuropeptide synthesis [4,5,6,7]. The activity of NGF is not restricted to embryonal life and although sensory neurons do not continue to be dependent on NGF for their survival, as sympathetic neurons do, the density of innervation [8,9], the expression of ion channels [10,11,12] and the synthesis of neurotransmitters and neuropeptides [13,14] are dynamically regulated by NGF. During adult life there is a basal production of NGF in the innervation field that is essential in regulating neuronal plasticity. There is a close correlation between the constitutive synthesis of NGF and the innervation density of adult tissues. The local production of NGF regulates cell body size, axonal sprouting and dendritic arborization [15,16]. In agreement with this, transgenic mice overexpressing NGF in epithelial structures show changes in neuronal phenotype associated with a striking increase in the number of axons, an altered distribution and enhanced branching of fibers in target organs [17,18,19,20].

So why refer to inflammation and immune responses in a review dedicated to nerve growth factor? The production of NGF is tightly controlled in all regions of the organism innervated by sensory and sympathetic neurons (i.e., the mucosae, skin and derma, internal organs) because its alteration profoundly modifies the organism’s physiology. Nevertheless, a significant increase in NGF synthesis in inflamed tissues has been described in patients and animal models of inflammatory diseases [21,22,23,24,25,26]. One of the effects of this enhanced production of NGF is the inflammatory pain associated with the NGF-induced expression of transient receptor potential vanilloid 1 (TRPV1) channels [27] and sodium channels [12,28] and the alteration of peripheral innervation [29,30]. Changes in NGF synthesis have a profound effect on neuron physiology [31] but, as the present review will show, they may also influence immune cell activity. This dual action of NGF on nervous and immune cells should not be surprising. The nervous and immune systems have identical functions: they are both responsible for maintaining homeostasis and for adapting the body to the environment [32]. In order to orchestrate strictly integrated responses, they need to maintain close anatomical connections and to share common chemical signals and specific receptors [33,34]. This direct bidirectional communication pathway enables a functional interaction between the two systems. Considering the well-known effects of NGF on peripheral neuron survival and its dynamic control of innervation and neuropeptide synthesis, together with the fact that NGF receptors are expressed in immune cells, it is intuitive that NGF can influence the activity of both systems. Indeed, a growing body of data indicates that NGF is a key molecule in the complex network of bidirectional signals between the nervous and immune systems.

## 2. NGF Is Enhanced during the Inflammatory Response

Studies on inflammatory and autoimmune diseases, which are characterized by an abnormal activation of immune cells and increased production of cytokines, have revealed a localized increase in NGF at the sites of inflammation. Enhanced NGF levels were initially found in the cerebrospinal fluid of multiple sclerosis patients, and it was shown that an increase in NGF closely follows the course of the disease [21]. The synovial fluids of patients with rheumatoid arthritis [22,23] are also characterized by an increased concentration of NGF, and its up-regulation in inflamed synovia was confirmed in studies on different animal models of induced arthritis [35,36]. Systemic Lupus Erythematosus (SLE) patients exhibit a significant increase in NGF concentration in the sera [24,25], which correlates with disease activity, and similar findings were obtained in studies of NZB/W mice, a spontaneous model of human SLE [37]. The list of inflammatory diseases characterized by an enhanced production of NGF in the inflamed tissues or in the blood is fairly long and includes diseases with different pathogenic mechanisms [38,39,40,41,42,43,44,45].

**Figure 1:**
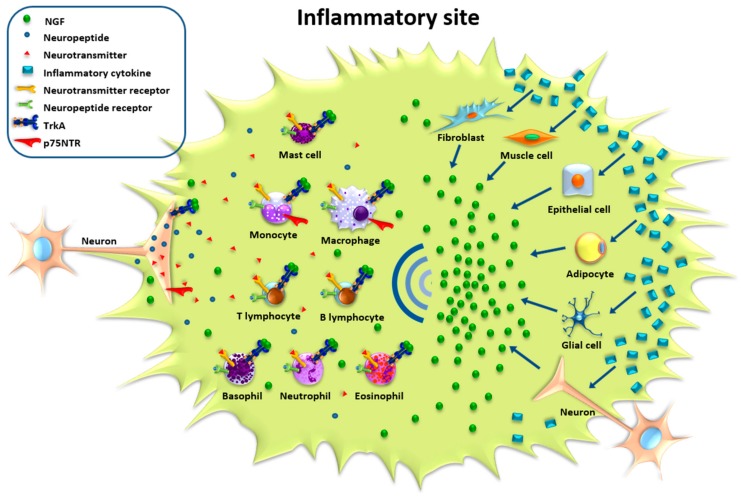
Direct and indirect effects of nerve growth factor (NGF) on inflammatory responses. At the site of inflammation, inflammatory cytokines induce (blue arrows) the production of NGF in different cell types, such as muscle cells, epithelial cells, fibroblasts, adipocytes, neurons, glia, and immune cells. The enhanced local production of NGF influences nerve fiber distribution and neuronal activity, inducing the synthesis and release of neuropeptides and neurotransmitters that have immunomodulatory effects. NGF receptors are also expressed on the membrane of immune cells and NGF can directly modulate the activity and functions of immune cells.

Thus, the normally low basal production of NGF is enormously up-regulated during inflammatory response. It is reasonable to hypothesize that, during the inflammatory process and because of tissue damage, there is a release of mediators that modify the local concentration of NGF. A number of studies have shown that cytokines involved in inflammation, such as IL-1β, TNF-α and IL-6, are promoters of NGF synthesis in a variety of cell types [46,47,48,49,50,51,52]. Inflammatory cytokines can induce the synthesis of NGF in neuronal and glial cells, as well as in epithelial, endothelial, connective and muscle cells (Figure 1). There are probably also other molecules that can up-regulate the basal production of NGF in tissue. For example, NGF production can be induced by prostaglandins [53,54] and histamine [55,56] in cultures of adipocytes, astrocytes and keratinocytes. Although the local alteration in NGF production seems to be correlated with inflammatory mediators, there are not many data demonstrating in vivo the specific cell types and mediators responsible for the enhanced production of NGF during inflammatory response in patients and in animal models of inflammatory diseases [57,58,59,60].

## 3. Expression of NGF Receptors in the Immune System

The distribution of NGF receptors in different areas of lymphoid organs suggests that local NGF production and its accumulation in afferent lymphoid vessels not only regulate the innervation of peripheral neurons but can also have an immunomodulatory activity. The expression of NGF and of the Tropomyosin Receptor Kinase A (TrkA) seems to be relevant for the differentiation of lymphoid organs, since structural abnormalities have been described in NGF receptor-deficient mouse lymphoid organs [61,62]. These findings suggest that NGF can regulate the differentiation of immune cells. The discovery of patients with mutations in the gene encoding TrkA [63] that cause an autosomal sensory neuropathy defined as congenital insensitivity to pain with anhidrosis (CIPA) provides further evidence that NGF can be involved in the regulation of the immune response in vivo. CIPA patients are characterized by neurological alterations and the absence of pain and sensation, but they also show reduced wound healing and modified functions of immune cells. They have altered immune mechanisms that are responsible for recurrent infections and inflammatory complications [64,65] that lead to chronic inflammatory responses [66].

### 3.1. Expression of NGF Receptors in Primary and Secondary Lymphoid Organs

The biological effects of NGF are mediated by its binding to two classes of receptors: p75 Neurotrophin Receptor (p75NTR), a 75 kDa glycoprotein that is a member of the TNF-receptor superfamily [67], and TrkA, a transmembrane tyrosine kinase of 140 kDa that is phosphorylated on tyrosine residues after binding to NGF [68]. TrkA is considered the NGF-specific receptor for its greater affinity and specificity for NGF compared with p75NTR which, in contrast, can also bind other neurotrophins with affinity similar to NGF. The majority of studies that have characterized TrkA and p75NTR expression and signaling in physiological and pathological conditions focused on neuronal cells and the peripheral and central nervous systems during development and in adult life [1,3,31,69,70].

The expression of these receptors is not restricted to the nervous system, but a number of studies have shown NGF receptor distribution in primary lymphoid organs (bone marrow, thymus and bursa of Fabricius), where the differentiation of immune cells from multipotent stem cells occurs (Table 1).

The thymus is a primary lymphoid organ in which T-cells differentiate, and it is active throughout childhood until puberty [71]. The thymus is composed of different types of cells (thymic epithelial cells, macrophages and dendritic cells), and thymocytes at various stages of T-cell differentiation move from the cortical to the medullar area. Both mRNA and protein levels of p75NTR have been detected in the thymus of mammals [72,73,74] as well as the expression of TrkA, although different TrkA isoforms have been described in the thymus [75,76]. The expression of TrkA specifically characterizes rat and human thymocytes [77,78,79]. TrkA is also expressed in epithelial subcapsular and medullar cells [73,75,80,81], in peripheral epithelial cells of Hassal’s bodies and in interdigitating reticular cells of the medulla [79]. The latter cells also express p75NTR, which is also expressed in periarteriolar macrophages, endothelial sinusal cells and nerve endings [79,80,81,82]. TrkA expression seems to be necessary for the normal development of the thymus, as demonstrated in TrkA-deficient mice, in which the thymus is smaller, with no clear delimitation between the cortex and the medulla, and the thymocyte density is lower [61].

The expression of TrkA and p75NTR is modulated in the rat embryo with mRNA levels of TrkA decreasing and of p75NTR increasing in the stroma of the thymus [78] during rat development. In the final stages of embryo development and in the early post-natal days the expression of TrkA and p75NTR in the rat thymus is maximal, and then declines with age [81] in parallel with age-dependent changes in medullar epithelial cells [82]. Interestingly, in normal human thymuses the epithelial cells exhibit a TrkA positive-p75NTR negative phenotype but a switch to a TrkA negative-p75NTR positive phenotype has been described in malignant epithelial cell tumors [73,83].

In bone marrow, a heterogeneous population of stromal cells provides the correct microenvironment for hematopoietic stem cell survival and differentiation in myeloid and lymphoid lineages. In human and rat, stromal cells with dendritic features expressing both TrkA and p75NTR appear in the fetal bone marrow before the hematopoietic activity begins [77,84]. CD34 positive hemopoietic stem cells also express NGF, p75NTR and TrkA, and in vitro this expression is maintained even in the absence of inducing factors [85,86,87].

Similarly to what has been described in bone marrow, there is also a gradient of expression of NGF receptors during embryo development in the bursa of Fabricius [88,89], the specific avian organ where the maturation and differentiation of B-cells occurs. During the post-hatching period, TrkA is expressed in epithelial cells of the follicle, in the interfollicular epithelium of the bursa of Fabricius and in blood vessels [90,91]. The local production of NGF seems to be involved in follicle differentiation in vivo, since NGF administration accelerates follicle formation in the chick embryo [91] and influences the survival of bursal cells [92].

The expression of NGF receptors also characterizes secondary lymphoid organs (spleen, lymph nodes and mucosa-associated lymphoid tissues) where antigen-presenting cells activate lymphocyte responses and effector cell differentiation.

The spleen is composed of two different tissues: the white pulp, responsible for the production and growth of immune and blood cells, and the red pulp, which filters microorganisms and cellular debris from the blood and removes older erythrocytes from circulation [93]. The expression of p75NTR and TrkA is localized primarily in the stroma of the spleen, with some expression in splenocytes [78] and in spleen mononuclear immunocompetent cells [77]. In rats, during spleen development, the level of p75NTR mRNA increases [78] and is expressed mainly by splenic nerve fibers and in a subpopulation of dendritic cells [62].

In lymph nodes and in mucosa-associated lymphoid tissues, TrkA is present in follicular dendritic cells, in blood vessel walls, in cryptic tonsillar epithelium and in several monocyte-derived cells including epithelioid and multinucleated Langhans’ cells and interdigitated reticular cells [79,94,95]. The expression of p75NTR has been described in follicular dendritic cells of lymphoid follicles, interdigitated reticular cells, periarteriolar macrophages, endothelial sinusal cells and nerve endings, and is also found in moderate levels in dendritic cells of tonsillar follicles [79,95,96].

The picture emerging from all these studies is that the expression of NGF receptors characterizes both stromal and immune progenitors and is finely regulated during development and post-natal life. Thus, the local production of NGF not only directly and actively modulates innervation patterns and fiber density in lymphoid organs [97], but can also be involved in the creation of the correct microenvironment that regulates the differentiation of myeloid and lymphoid precursors [98]. A few in vitro studies have focused specifically on the response of stromal cells to NGF. After treatment of stromal cells derived from human bone marrow with NGF, microarray analysis demonstrated changes in cytokine gene expression in response to NGF that can influence hematopoiesis [99]. In thymic epithelial cells, the addition of NGF enhanced the expression of adhesion molecules needed for thymocyte-thymic epithelia interaction and up-regulated thymopoietic factor expression (SDF-1, IL-7, GM-CSF) [100].

### 3.2. Expression of NGF Receptors in Immune Cells

The expression of TrkA characterizes hemopoietic stem cells and is highest in the more undifferentiated cells, declining during lineage differentiation [85,86]. These stem cells can also produce their own NGF in an autocrine fashion, which seems necessary to increase the long-term survival and regulate differentiation of human hemopoietic cells [101,102,103,104]. As demonstrated in human cord blood cells, a gradient of TrkA and NGF expression exists that is highest in CD34-positive cells, reduced in cord blood mononuclear cells and minimal in mononuclear cells isolated from adult peripheral blood, and further declines with age [86,105]. TrkA expression is maintained during lineage differentiation and has been demonstrated in purified blood mononuclear cells, thymocytes, human B- and T-lymphocytes, monocytes, mast cells, basophils, eosinophils and neutrophils [86,105,106,107,108,109,110,111,112].

In B- and T-lymphocytes, undifferentiated monocytes and macrophages, the basal expression of TrkA is strongly up-regulated after antigenic or inflammatory stimulation when functional activity is necessary [106,107,108,113,114]. TrkA is poorly expressed in normal B- and T-lymphocytes, but is up-regulated in several B-cell lymphoma subtypes, anaplastic large cell lymphomas and Reed-Sternberg cells [79].

The expression of p75NTR has been described for many immune cells but there is still little information concerning changes in p75NTR during immune cell differentiation and activation [115,116]. A recent study on monocytes demonstrated that p75NTR expression is fairly low compared with TrkA in a normal resting condition [117]. Monocytes lose TrkA expression and only p75NTR expression is maintained [118] when these cells differentiate in dendritic cells, the antigen-presenting cells that interact closely with T-cells to form the immunological synapse and activate T-cell effector responses. This dynamic regulation of NGF receptor expression during immune cell differentiation and response suggests a differential need for NGF of immune cell populations, depending on their state of maturity and functional activity.

## 4. NGF and Its Direct and Indirect Effects on Immune Response

### 4.1. Indirect Action

The innervation of primary and secondary lymphoid organs constitutes the anatomical link between the nervous and immune systems. The integrity of this connection is essential to maintain a regulatory function of the nervous system on immune response: experiments of chemical sympathectomy, denervation of lymphoid organs or vagotomy [33,34,119] have clearly demonstrated alterations in the proliferation and responsiveness of immune cells and a modified migration. Nerve fibers end in the parenchyma in close contact with immune cells, and in this “neuroimmune junction” [120] the release of neurotransmitters from nerve endings can stimulate specific receptors present on immune cells affecting their functions. Muscarinic, nicotinic and adrenergic receptors are expressed on immune cells [121,122,123,124], and the release of neurotransmitters from nerve endings can finely tune the innate and adaptive immune responses by activating either pro- or anti-inflammatory pathways [33,34,119,120,121,122,123,124]. Sensory neuropeptides can also activate specific responses by binding to their specific receptors expressed on immune cells. The release of neuropeptides is a pivotal event in neurogenic inflammation, a well-studied neuroimmune mechanism involved in the development of inflammatory diseases such as rheumatoid arthritis, psoriasis, allergy and asthma [125,126]. Neuropeptides, including substance P (SP) and calcitonin gene-related peptide (CGRP), released from nerve endings induce vasodilatation and plasma extravasation, promote leukocyte chemotaxis and phagocytosis, and can directly affect the release of cytokines and inflammatory mediators from immune cells [127]. Thus different types of immune cell populations express “neuronal” receptors and respond to neuropeptide and neurotransmitters, indicating that peripheral neurons are an integral part of the local effector systems involved in the inflammatory responses to tissue irritation and injury.

Through its effects on neuronal cells, NGF can indirectly regulate the immune response in a variety of manners. It can affect neuronal plasticity in the inflamed tissue by increasing innervation density, axonal terminal sprouting and dendritic arborization.

In inflamed tissues there is a marked alteration in nerve fiber distribution, with an increased terminal sprouting leading to hyper-innervation of the injured tissues.

Inflammation leads to an increased sensitivity to pain and intense stimulation and firing of the nociceptors, which induces the release of neuropeptides and neurotransmitters from nerve endings in close contact with the cell membranes of keratinocytes, mast cells, macrophages, Langerhans cells and endothelial cells [33,34,119,120,121,122,123,124,125,126,127,128,129] (Figure 1).

NGF not only influences fiber density and dendritic sprouting in the inflamed tissues, but may also affect the local immune response by directly regulating the production of neurotransmitters and neuropeptides, which, as a growing body of findings is showing, have direct effects on immune cells and are involved in the development of many inflammatory diseases [4,5,6,7,8,9,13,14,15,16,19,20,29,30,97] (Figure 1). The increased concentration of NGF in inflamed tissues can regulate the expression of neuropeptides and neurotransmitters in peripheral neurons by directly acting on their promoters or by regulating the activity of neurotransmitter-producing enzymes. For example, in sympathetic neurons the production of norepinephrine is controlled by NGF through induction of the transcription of tyrosine hydroxylase, the rate-limiting enzyme of the catecholamine biosynthetic pathway [4]. In sensory neurons, however, NGF directly controls the expression of SP and CGRP. Thus, peripheral neurons exposed to enhanced concentrations of NGF in the inflamed target tissue modify the synthesis of neuropeptides and neurotransmitters and can alter their phenotype so that different types of neurotransmitters can be produced and stored in nerve endings. [6,13,14,29,130,131]. Many recent studies have focused on the release of acetylcholine and the activation of parasympathetic nerves because of their ability to inhibit inflammation and activate anti-inflammatory pathways [132]. Cholinergic neurons in the central nervous system are dependent on NGF for their survival and phenotype maintenance. Although NGF can regulate acetylcholine synthesis by increasing choline acetyltransferase activity [133], it is not clear whether it can influence parasympathetic neuron functions. Recent studies have shown that NGF can activate a parasympathetic tone. In mouse airway parasympathetic ganglia, the cholinergic neurons expressed TrkA and exposure to NGF potentiated synaptic transmission, enhancing their excitability [134,135]. In vivo administration of NGF altered dendritic length and sprouting in the bronchial ganglionic neurons of guinea pigs [134]. All together, these data suggest that NGF can influence parasympathetic neuron activity and contribute to activate regulatory cholinergic anti-inflammatory pathways responsible for the inhibition of pro-inflammatory cytokine release in the “inflammatory reflex” [136].

### 4.2. Direct Action

The presence of NGF receptors on cells other than neuronal cells opens new perspectives regarding the range of action of NGF. The first indication of an immunoregulatory effect of NGF was observed in 1977 by Aloe and Levi-Montalcini, who showed that injecting neonatal rats with NGF resulted in an increase in the number and size of mast cells [137]. It has now been amply demonstrated that TrkA and p75NTR are present on the surface of immune cells, as well as how their expression is modulated depending on the state of activation of the cells [108,111,114]. In vitro studies using purified immune cell populations have demonstrated a number of actions that can be ascribed to NGF and are summarized in Table 2.

NGF regulates the survival of different immune cells in a similar manner to its effect in neurons. It promotes the survival of hematopoietic stem cells [85], eosinophils [151], neutrophils [152,153], mast cells [149,150], B-cells [107] and monocytes [118]. The activation of TrkA pathways seems to inhibit apoptosis through regulation of the expression of Bcl-2, Bcl-xl and Bfl-1 [101,107,149,150]. Using different cell populations, it has been shown in vitro that NGF alone does not induce the synthesis of chemokines or cytokines. On the contrary, after inflammatory stimulation, the addition of NGF potentiates endogenous responses, inducing the release of cytokines and inflammatory mediators [138,139,140,141,142,143,144,145,146,147,151,153,154,162,166]. NGF also induces in immune cells the synthesis of neuropeptides with immunomodulatory functions: i.e., neuropeptide Y in T-cells [168], calcitonin gene-related peptide in monocytes [169], and B-cells [170]. Cells of the innate immune system respond to NGF by activating a variety of responses: NGF activates chemotaxis [148,164,165,171], stimulates the phagocytosis of neutrophils [153] and macrophages [164,165], enhances the cytotoxic activity of eosinophils [151], and induces the degranulation of mast-cells [146,149].

NGF also potentiates the proliferative response of T- and B-cells to several mitogens [155] and modulates B-cell-mediated immune responses and immunoglobulin secretion [107,157,158,159,160], promoting the differentiation of B-cells into immunoglobulin-secreting plasma cells [161].

## 5. In Vivo Inflammatory and Anti-Inflammatory Mechanisms and the Roles of NGF and TrkA

Taken together, the available in vitro data suggest that NGF potentiates the activity of innate immune cells and influences B- and T-cell mediated responses. However, the mechanism by which NGF modulates the immune response in vivo is still not fully understood.

At present, despite the well-documented increase in NGF concentrations measured in the inflamed tissues of many inflammatory diseases, little is known of the effects of increased levels of NGF or of the activation of its receptors during inflammatory response. Currently available data suggest that the action of NGF is more complex in vivo than it is in vitro.

In animal models and in humans, the administration of NGF alone without a more specific inflammatory stimulus, while inducing activation of local sensory hypersensitivity, does not activate any inflammatory response [172,173,174,175].

However, the intraventricular administration of NGF after the induction of experimental autoimmune encephalomyelitis (EAE) in marmoset [176] or mouse models [177,178] delayed disease onset and decreased disease severity, preventing the full development of brain lesions. Reduced inflammatory infiltrate and demyelination were found in the brains of NGF-treated EAE animals, associated with a reduced production of interferon-gamma but with an enhanced production of IL-10 [176,178]. Similarly, when myelin basic protein-specific CD4+ T-cell clones were used to induce EAE, the contemporary injection of transfected T-clones encoding for NGF resulted in mild disease with minor symptoms [177]. Consistent with this observation, in vivo NGF deprivation in rats with EAE resulted in an increased brain inflammation and more severe clinical features [179]. Interestingly, it has been demonstrated in some animal models that the well-known increase in NGF production associated with inflammation remained elevated until the inflammatory response subsided [180]. Neutralization with anti-NGF antibodies of the enhanced production of NGF increased the disease symptoms with a higher number of infiltrating neutrophils and macrophages and more extended gut lesions in animal models of colitis [181]. Also, in contact hypersensitivity animal models, the neutralization of endogenous NGF resulted in a loss of inhibition of the systemic response [60].

Taken all together, the results obtained in different models of inflammation indicate that in vivo NGF administration ameliorates tissue inflammation, suggesting a direct influence of NGF on antigen presentation, down-regulation of inflammatory cytokines and up-regulation of the anti-inflammatory cytokine IL-10.

Although some discrepancies exist in the effects of NGF in vitro and in vivo, it seems reasonable to hypothesize that NGF is part of an endogenous mechanism that, while activating innate immune responses, also activates pathways necessary to dampen inflammatory response and limit tissue damage.

**Figure 2:**
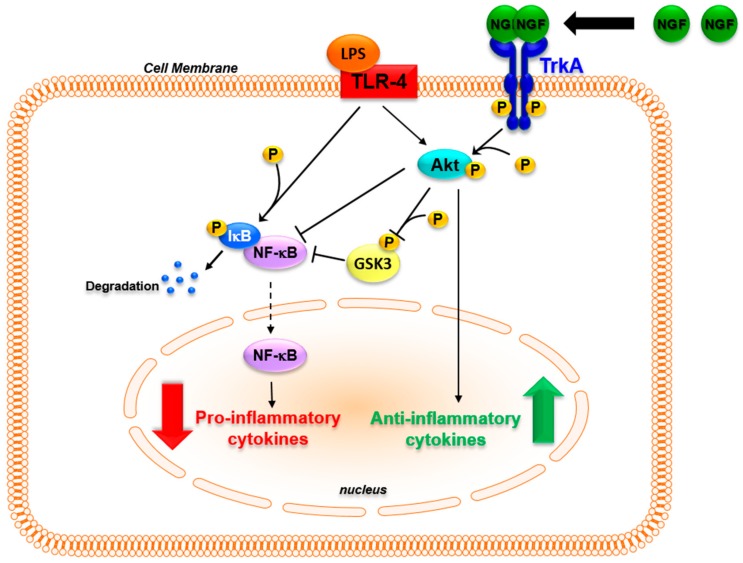
TrkA activation promotes anti-inflammatory pathways. In human monocytes the expression of TrkA is enhanced when TLR4 is activated. Auto-phosphorylation of TrkA induced by NGF binding activates intracellular pathways that influence the downstream signaling of TLR4. The NGF-induced phosphorylation of Akt inhibits NF-κB translocation in the nucleus. The inhibitory phosphorylation of GSK3 induced by Akt further prevents NF-κB activation, and the NF-κB-dependent transcription of pro-inflammatory cytokine genes. Concomitantly, NGF activation of the PI3K/Akt pathway induces the expression of IL-10 and IL-1 receptor antagonist (IL-1ra), promoting a net anti-inflammatory effect. Black bold arrow shows dimerization of NGF and binding to TrkA. Red and green bold arrows show respectively decrease and increase of cytokine levels. Black slim arrows and black T bar show respectively activation and inhibition of molecular pathways. Dotted line arrow show nuclear translocation.

It should be kept in mind that inflammation is an adaptive response whose main aim is to restore homeostasis [182]. One key aspect of the inflammatory response and of its resolution is the parallel activation of pathways leading to inflammatory cytokine production that activates innate and adaptive immune responses, and of pathways that induce anti-inflammatory cytokines such as IL-10 and IL-1 receptor antagonists in order to avoid tissue destruction [183,184]. The possibility that NGF may be involved in these physiological mechanisms is supported by recent findings. In monocytes, inflammatory stimuli, while activating pro-inflammatory responses through Toll-like receptors (TLR), up-regulate the expression of the NGF receptor TrkA [108,114,117,118]. In TLR-activated monocytes, the binding of NGF to TrkA influences TLR signaling, decreasing NF-κB nuclear translocation and inhibiting glycogen synthase kinase 3 (GSK3) activity, which leads to a decreased production of inflammatory cytokines [117,185,186]. Moreover, TrkA activation further enhances the TLR-induced activation of the PI3K/Akt pathway. This pathway is one of the main anti-inflammatory pathways activated to reduce TLR ligand-induced inflammatory responses [184]. The activation of TrkA that followed TLR activation resulted in an increased production of anti-inflammatory cytokines [117,187]. Thus, the binding of NGF to TrkA can activate distinct pathways that influence the intracellular signaling activated after the recognition of inflammatory stimuli with an overall anti-inflammatory action, thus influencing the production of cytokines that are pivotal in orchestrating innate and adaptive immune responses (Figure 2). The activation of TrkA might be one of the signals that, when the immune system is activated, is involved in endogenous regulatory feed-back mechanisms enhancing the activation of anti-inflammatory pathways. This might explain the in vivo findings, in which the addition of NGF tilted the balance of the pro- and anti-inflammatory pathways that are simultaneously activated during inflammatory response, potentiating the release of anti-inflammatory mediators such as IL-10 [183,184,187]. Consequently, changes in TrkA expression levels may affect the physiological anti-inflammatory mechanism mediated by NGF. Consistent with this hypothesis, patients with chronic arthritis have a marked down-regulation of TrkA expression in blood and synovial mononuclear cells when compared with healthy donors [117]. In ex vivo experiments, the addition of NGF to TLR-activated mononuclear cells of arthritis patients, which are characterized by a significant decrease in TrkA expression, failed to reduce the production of IL-6 that was instead observed after NGF treatment in healthy donor cells expressing high TrkA levels [117]. The TrkA imbalance found in arthritis patients might prevent NGF from activating the regulatory anti-inflammatory feed-back mechanisms, thus contributing to the development and maintenance of chronic inflammation. Further studies aimed at evaluating TrkA expression in other inflammatory diseases may help us to understand whether this is a general mechanism in chronic inflammatory diseases.

## 6. Conclusions

A growing body of data suggests that NGF is a key regulator of the cross-talk between the immune and nervous systems. While the effects of NGF on neuronal cell survival, differentiation and phenotype maintenance are fairly well-known, the effects of NGF on immune cells have still not been completely defined. Further investigations are needed to establish why NGF is synthesized in vivo in the inflamed tissue and how the activation of its receptors can influence the cellular and molecular mechanisms triggered during the inflammatory response. What has already been demonstrated in neuronal cells and is now emerging in studies on immune cells and inflammatory diseases is that NGF definitely controls a multitude of effects, some of them even contradictory. The diverse biological activities of NGF on immune cell populations can be explained by considering the differential expression of NGF receptors during different states of cell differentiation and activation. The complexity of NGF action and its signaling can be better understood if we consider them as being correlated with the need for differing types of regulation in a variety of immune cell types in order to maintain correct homeostasis. Alteration in NGF synthesis and modification of the expression of its receptors may influence physiological responses and be involved in the pathogenesis of inflammatory diseases.

A better understanding of the physiological role of NGF and its receptors in regulating immune responses could be of groundbreaking importance with translational implications for human diseases.

## Figures and Tables

**Table 1:** Distribution of NGF in primary and secondary lymphoid organs.

NGF Receptor Distribution in Lymphoid Organs			
Tissue		TrkA	p75NTR
Primary Lymphoid Organs	Thymus	thymocytes [77,78,79]	interdigitating reticular cells of the medulla [79];
		epithelial subcapsular and medullar cells [73,75,80,81]	periarteriolar macrophages [79,80,81,82];
		peripheral epithelial cells of Hassal’s bodies [79]	endothelial sinusal cells and nerve endings [72,79,80,81,82];
		interdigitating reticular cells of the medulla [79]	-
	Bone marrow	stromal cells with dendritic features [77,84]	stromal cells with dendritic features [77,84];
		CD34 positive hemopoietic stem cells [85,86,87]	CD34 positive hemopoietic stem cells [85,86,87];
	Bursa of fabricius	epithelial cells of the follicle [90,91]	bursa of Fabricius of chick embryo [88];
		interfollicular epithelium [90,91]	
		blood vessels [90,91]	
Secondary Lymphoid Organs	Spleen	stroma of the spleen and splenocytes [78]	stroma of the spleen and splenocytes [78];
		spleen mononuclear immunocompetent cells [77]	spleen mononuclear immunocompetent cells [77];
	Lymph nodes and mucosa-associated lymphoid tissues	follicular dendritic cells [79,94,95]	follicular dendritic cells [79,94,95];
		blood vessel walls [79,94,95]	blood vessel walls [79,94,95];
		cryptic tonsillar epithelium [79,94,95]	cryptic tonsillar epithelium [79,94,95];
		monocyte-derived cells [79,94,95]	monocyte-derived cells [79,94,95];
		interdigitated reticular cells [79,94,95]	interdigitated reticular cells [79,94,95].

**Table 2:** Multiple effects of NGF on purified immune cells.

Effect of NGF Stimulation	
Activated basophils	↑ leukotriene and cytokine synthesis [110,138,139,140]
	↑ histamine release [141]
	↑ response to IgE [141,142]
Immature mast cells	↑ tryptase and IgE receptors [143]
Mature mast cells	↑ cyclooxygenase2 (COX2) and prostaglandin D2 [144]
	↑ IL-6 induction [145]
	↑ histamine release [146,147]
	↑ chemotaxis [148]
	↑ survival (by suppressing apoptosis) [149,150]
Eosinophils	↓ suppression of leukotriene formation [140]
	↑ IL-4 production [111]
	↑ peroxidase release and cytotoxic activity [151]
	↑ survival (by suppressing apoptosis) [151]
Neutrophils	↑ survival (by suppressing apoptosis) [152]
	↑ superoxide production and phagocytosis [153,154]
B-cells	↑ proliferative response [155,156]
	↑ IL-2 receptors [156]
	influences the production of IgM and IgG [157,158,159,160]
	↑ survival of memory B-cells [107]
	↑differentiation of B-cells into immunoglobulin-secreting plasma cells [157]
	influences plasma cell survival [161]
T-cells	↑ proliferative response [155]
	↑ cytokine expression [162]
Monocytes/macrophages	protection from apoptosis, by inducing the anti-apoptotic proteins Bcl-2, Bcl-xl and Bfl-1 [118]
	↑ CXCR4 expression and chemotactic response [163,164]
	↑ phagocytosis, enhanced parasite-killing activity and IL-1β [165]
	↑ TNF-α, IL-8 secretion [154,166]
Dendritic cells	↑ maturation of dendritic cells and secretion of inflammatory cytokines [167]
	↑ IL-6 release in allergic patients;
	↑ IL-10 release in healthy controls [167]

Footnote: ↑ = increase; ↓ = decrease.
